# Association of complete blood count parameters, d‐dimer, and soluble P‐selectin with risk of arterial thromboembolism in patients with cancer

**DOI:** 10.1111/jth.14484

**Published:** 2019-06-17

**Authors:** Ella Grilz, Christine Marosi, Oliver Königsbrügge, Julia Riedl, Florian Posch, Wolfgang Lamm, Irene M. Lang, Ingrid Pabinger, Cihan Ay

**Affiliations:** ^1^ Clinical Division of Hematology and Hemostaseology Department of Medicine I Comprehensive Cancer Center Vienna Medical University of Vienna Vienna Austria; ^2^ Clinical Division of Oncology Department of Medicine I Comprehensive Cancer Center Vienna Medical University of Vienna Vienna Austria; ^3^ Division of Oncology Department of Internal Medicine Medical University of Graz Graz Austria; ^4^ Clinical Division of Cardiology Department of Medicine II Medical University of Vienna Vienna Austria

**Keywords:** arterial occlusive diseases, biomarkers, blood cell count, neoplasms, thrombosis

## Abstract

**Background:**

Patients with cancer are at risk of developing arterial thromboembolism (ATE). With the prevalence of cancer and cardiovascular diseases on the rise, the identification of risk factors for ATE in patients with cancer is of emerging importance.

**Objectives:**

As data on the association of potential biomarkers with risk of ATE in patients with cancer are scarce, we conducted a cohort study with the aim to identify blood‐based biomarkers for ATE risk prediction in patients with cancer.

**Patients/Methods:**

Overall, 1883 patients with newly diagnosed cancer or progressive disease after complete or partial remission were included and followed for 2 years. Venous blood was drawn at study inclusion for measurement of complete blood count parameters, total cholesterol, d‐dimer, and soluble P‐selectin (sP‐selectin) levels.

**Results:**

The 2‐year cumulative incidence of ATE was 2.5%. In univariable analysis, red cell distribution width (subdistribution hazard ratio (SHR) per doubling: 4.4, 95% CI: 1.4‐14.1), leukocyte count (1.2, 1.1‐1.5), neutrophil count (1.6, 1.1‐2.3), and sP‐selectin levels (1.9, 1.3‐2.7) were associated with risk of ATE in patients with cancer; d‐dimer was not associated with the risk of ATE (1.1, 0.9‐1.4). After adjustment for age, sex, and smoking status the association prevailed for the neutrophil count (adjusted [adj.] SHR per doubling: 1.6, 1.1‐2.4), and sP‐selectin levels (1.8, 1.2‐2.8).

**Conclusions:**

An elevated absolute neutrophil count and higher sP‐selectin levels were associated with an increased risk of ATE in patients with cancer. Their role for predicting cancer‐related ATE needs to be validated in further studies.

**Essentials**

- Patients with cancer are at risk of developing arterial thromboembolism (ATE).
- Here we present the results of a cohort study in 1883 patients with cancer.
- An elevated neutrophil count was associated with a higher risk of ATE in patients with cancer.
- Higher soluble P‐selectin levels were associated with higher ATE risk in patients with cancer.

## 1 INTRODUCTION

Since the interaction of cancer and hemostasis was recognized in the nineteenth century, subsequent research has focused mainly on cancer‐associated venous thromboembolism (VTE).^1^, ^2^, ^3^, ^4^, ^5^, ^6^ However, thrombosis can also occur in the arterial system as a consequence of cancer‐induced hypercoagulability. In contrast to cancer‐associated VTE, the association between cancer and arterial thromboembolism (ATE) has been just recently established.^7^, ^8^, ^9^

While many risk factors and biomarkers have been identified for cancer‐associated VTE, data regarding potential biomarkers for cancer‐associated ATE are scarce.^10^, ^11^, ^12^, ^13^, ^14^, ^15^, ^16^, ^17^, ^18^ For example, d‐dimer ‐ which is formed after plasmin‐mediated fibrin polymer degradation ‐ is a well‐known biomarker for VTE in the oncologic and non‐oncologic settings.^19^ Furthermore, d‐dimer is also a predictor for ATE in patients without cancer.^20^, ^21^, ^22^, ^23^, ^24^ Recently, Ryu et al reported an association between d‐dimer levels and the risk of stroke in patients with cancer.^25^ Another molecule that has attracted attention as a biomarker for both VTE and ATE is P‐selectin, which is a cell adhesion molecule, mainly stored in endothelial cells and platelets. Upon activation of these cells P‐selectin is translocated to the cell surface and then released to the plasma as soluble form (sP‐selectin). The P‐selectin expression on platelets is reflecting platelet activation. Moreover, the binding of P‐selectin to its main receptor, P‐selectin glycoprotein ligand‐1, triggers thrombus growth and fibrin formation and thereby leads to a hypercoagulable state.^19^ Prior studies revealed an association of sP‐selectin with VTE and ATE in populations without cancer.^26^, ^27^, ^28^, ^29^, ^30^ Elevated sP‐selectin levels have also been identified to predict cancer‐associated VTE.^31^ The role of d‐dimer and sP‐selectin levels for prediction of ATE in patients with cancer is currently unknown.

As the prevalence of cardiovascular diseases and cancer increases in an aging population, a better understanding of the interrelation between cardiovascular diseases and cancer is of growing socioeconomic and clinical importance.^32^, ^33^, ^34^, ^35^ Therefore, we conducted a cohort study with the aim to identify blood‐based biomarkers (i.e. blood count parameters, total cholesterol, d‐dimer, and sP‐selectin levels) for ATE risk prediction in patients with cancer.

## 2 METHODS

### 2.1 Study design and population

For this analysis we used data from the Vienna Cancer and Thrombosis Study (CATS). In 2003, CATS was initiated at the Medical University of Vienna, Austria, after approval by the local ethics committee (EC number: 126/2003; ethic-kom@meduniwien.ac.at), and is conducted in accordance with the Declaration of Helsinki. CATS is a single‐center cohort study with a baseline biobank aiming to identify biomarkers for venous thrombotic risk in the oncologic setting. Adult patients (≥18 years) with newly diagnosed cancer or progressive disease after complete or partial remission were included in CATS. After written informed consent, patients were followed for a maximum duration of 2 years, until withdrawal of consent, occurrence of VTE, loss of follow‐up, or death, whichever occurred first. Patients’ follow‐up was carried out by postal letter or phone every 3 months. In addition, if necessary, the family doctor or relatives were interviewed by telephone.

Patients who had undergone chemotherapy treatment within the last 3 months prior to study inclusion or radiotherapy or surgery within the last 2 weeks prior to study inclusion were ineligible. Furthermore, patients who experienced thrombosis within 3 months before study inclusion or had an indication for long‐term anticoagulation were not included in CATS. However, patients with platelet aggregation inhibitors (e.g. acetylsalicylic acid) were eligible. More detailed information about the study design, exclusion and inclusion criteria, and study procedures has been reported previously.^31^

Between 17 October 2003 and 30 September 2013, 2004 patients were recruited in CATS. Overall, 121 patients had to be excluded, because they did not fulfill inclusion criteria (*n* = 35) or they had exclusion criteria (*n* = 60) after reevaluation, no laboratory material was available (*n* = 7), no follow‐up was available (*n* = 17), or patients withdrew consent (*n* = 2). Thus, overall 1883 patients with active cancer were included in the present analysis.

### 2.2 Outcome measurement

The predefined primary outcome of CATS was VTE, while data on ATE were collected as a comorbid condition during the study period. For the present analysis objectively confirmed symptomatic ATE was the primary endpoint. The ATE was defined as a composite of myocardial infarction, ischemic stroke, and peripheral arterial occlusion with an interventional procedure. A panel of experts in the field of cardiology, neurology, and vascular medicine adjudicated all ATEs. More detailed information regarding the definition and adjudication of ATE has been reported previously.^36^

### 2.3 Blood sampling and laboratory analyses

On the day of enrollment blood samples were collected via sterile and atraumatic venipuncture in Vacutainer K3‐EDTA tubes, Vacutainer citrate tubes (containing 1:10 volume sodium citrate solution at 0.129 mmol/L), and Vacutainer Z serum separator clot activator tubes (all Vacuette; Greiner‐Bio One, Kremsmünster, Austria).

Erythrocyte count, hemoglobin, hematocrit, mean corpuscular volume, mean corpuscular hemoglobin, mean corpuscular hemoglobin concentration, red cell distribution width, platelet count, leukocyte count, neutrophil count, and lymphocyte count were routinely measured within 2 hours from blood sampling in an accredited process using the Sysmex XE‐5000 hematology analyzer. Total cholesterol was routinely measured using an enzymatic‐colorimetric CHOD‐PAP method. In addition, citrated blood samples were centrifuged to obtain platelet‐poor‐plasma and aliquots were stored at −80°C for serial measurement of d‐dimer and sP‐selectin as described in detail in prior publications.^31^, ^37^

### 2.4 Statistical analysis

Count data were summarized as absolute and relative frequencies. Continuous variables were described via medians and interquartile range (25th to 75th percentile). The reverse Kaplan‐Meier method was used to estimate median follow‐up time.^38^

A competing risk estimator with 95% confidence intervals (95% CIs) was used to calculate the cumulative incidence of ATE.^39^ The ATE incidences between groups were compared with Gray′s test.^40^ Univariable and multivariable Fine and Gray competing risk regression models were used to estimate SHRs of ATE. The SHRs are reported per doubling of the parameter (i.e. 1‐unit increase of the log2‐transformed variable). Venous thromboembolism and death from any cause were considered competing events.^41^ Age, male sex, and smoking were previously identified as predictors for ATE in patients with cancer.^36^ Therefore, we prespecified these three parameters as covariables for all multivariable analyses.

For graphical illustration of ATE risk over time and in the absence of validated cutoffs we empirically dichotomized ATE biomarkers into binary variables. As in previous studies, we chose the 75th percentile of their distribution in the total study population before analyses we performed.^4^, ^42^ Because of the relatively low incidence of arterial events in prior publications and the arbitrary nature of cutoffs we considered the possibility that between‐group comparison may not reach statistical significance when dichotomized at an empirically set cutoff.^7^ Therefore, statistically optimal cutoff points were determined during the analyses using Youden′s index of the receiver operating curve.^43^ First, we computed sensitivity and specificity for ATE at each possible biomarker cutoff, and then we calculated Youden'sindex for each cutoff point as sensitivity + specificity‐1. Then, we treated patients as having an elevated ATE biomarker in case their biomarker measurement was larger than the biomarker measurement with the highest Youden's index.

SPSS 25 (SPSS Inc., Chicago, IL) and STATA 15.0 (STATA Corp., Houston, TX) were used to perform all statistical analyses. Statistical significance was predefined as a 95% confidence interval that does not include the null hypothesis value.

## 3 RESULTS

### 3.1 Population

Overall, 1883 patients (861 [45.7%] female) with active cancer and a median age of 61 years were followed for 670 days in median. In 1388 (73.7%) patients cancer was newly diagnosed, and 495 (26.3%) patients had progressive disease after complete or partial remission. Detailed information about the study cohort is given in Table 1. Overall, 757 (40.2%) patients died during the observation period and 187 (8.3%) developed VTE.

**Table 1:** Characteristics of the total study cohort and of patients who developed arterial thromboembolism

	All patients	ATE during follow‐up^a^
	*n* = 1883	*n* = 48 (2.5%)
Median age at study entry, years (IQR)	61 (52‐68)	66 (60‐69)
Median body mass index (IQR)	25.1 (22.3‐28.3)	26.3 (23.2‐29.0)
Sex, *n*		
Female	861	11 (1.3%)
Male	1022	37 (3.6%)
Site of cancer, *n*		
Lung	321	15 (4.7%)
Breast	276	0 (0.0%)
Lymphoma	265	5 (1.9%)
Brain	249	7 (2.8%)
Colon/rectum	186	3 (1.6%)
Prostate	157	8 (5.1%)
Pancreas	133	2 (1.5%)
Stomach	65	2 (3.1%)
Multiple myeloma	50	0 (0.0%)
Kidney	45	4 (8.9%)
Others	136	2 (2.5%)
Cancer stage		
Localized	609	19 (3.1%)
Distant metastasis	629	14 (2.2%)
Not classifiable^b^	565	12 (2.1%)
Unknown	80	3 (3.8%)
Smoking status		
Smoker	547	19 (3.5%)
Ex‐smoker (>1 year non‐smoker)	314	12 (3.8%)
Non‐smoker	868	16 (1.8%)
History of VTE^c^, *n*	98	2 (2.2%)
Known atherosclerotic/cardiovascular disease at study entry, *n*	159	12 (7.5%)
Platelet inhibitor use at study entry, *n*	280	22 (7.9%)
Median erythrocyte count, 10^12^/L (IQR)	4.4 (4.0‐4.7)	4.4 (4.1‐4.7)
Median hemoglobin, g/dL (IQR)	13.1 (11.8‐14.1)	13.1 (12.1‐14.1)
Median hematocrit, % (IQR)	38.9 (35.4‐41.5)	38.6 (36.3‐42.2)
Median mean corpuscular volume, fL (IQR)	88.5 (85.4‐91.7)	88.6 (85.3‐92.2)
Median mean corpuscular hemoglobin, pg (IQR)	29.9 (28.6‐31.0)	30.1 (28.2‐31.2)
Median mean corpuscular hemoglobin concentration, g/dL (IQR)	33.7 (32.9‐34.4)	33.5 (32.8‐34.3)
Median red cell distribution width, % (IQR)	13.8 (13.1‐14.6)	14.2 (13.5‐15.6)
Median platelet count, 10^9^/L (IQR)	248.5 (197.0‐309.0)	247.0 (201.0‐294.0)
Median mean platelet volume, fL (IQR)	10.2 (9.6‐10.8)	10.0 (9.6‐11.1)
Median leukocyte count, 10^9^/L (IQR)	7.2 (5.7‐9.6)	8.9 (6.5‐10.5)
Median neutrophil count, 10^9^/L (IQR)	4.9 (3.5‐6.7)	5.7 (4.3‐7.2)
Median lymphocyte count, 10^9^/L (IQR)	1.4 (1.0‐1.8)	1.4 (1.1‐1.8)
Median cholesterol level, mg/dL (IQR)	206.0 (173.0‐240.0)	189.5 (162.0‐244.0)
Median d‐dimer level, μg/mL (IQR)	0.7 (0.4‐1.4)	1.0 (0.3‐2.4)
Median sP‐selectin level, ng/mL (IQR)	38.5 (29.4‐49.7)	46.3 (34.1‐53.8)

Note

Data were missing for hemoglobin (*n* = 5), platelet count (*n* = 5), body mass index (*n* = 6), leukocyte count (*n* = 6), hematocrit (*n* = 8), mean corpuscular volume (*n* = 8), mean corpuscular hemoglobin (*n* = 8), mean corpuscular hemoglobin concentration (*n* = 8), sP‐selectin (*n* = 8), red cell distribution width (*n* = 10), erythrocyte count (*n* = 11), mean platelet volume (*n* = 37), d‐dimer (*n* = 61), neutrophil count (*n* = 148), lymphocyte count (n = 148), smoking status (*n* = 154), and cholesterol level (*n* = 579).

Abbreviations: ATE, arterial thromboembolism; IQR, interquartile range (i.e. 25^th^ to 75^th^ percentile); sP‐selectin, soluble P‐selectin; VTE, venous thromboembolism.

a Percentages are related to numbers given in the first column of the same line.

Brain and hematologic tumors.

VTE that had occurred at least 3 months or more before study inclusion.

### 3.2 Risk of arterial thromboembolism and mortality

During the 2‐year observational period 48 (2.5%) patients developed ATE. The most common index event was myocardial infarction (*n* = 20, 41.7%). Other ATE events were major and minor stroke in 13 (27.1%) and 3 (0.6%) cases, as well as peripheral arterial occlusion in 12 (25.0%) patients, respectively. The cumulative 3‐month, 6‐month, 12‐month, and 24‐month ATE risk was 0.9% (95% CI: 0.5‐1.4), 1.1% (0.7‐1.7), 1.7% (1.2‐2.4), and 2.6% (2.0‐3.4), respectively.

### 3.3 Association of blood count parameters with ATE risk

In competing risk regression analysis, erythrocyte count was not associated with the risk of ATE in patients with cancer (SHR per doubling: 1.5, 95% CI: 0.4‐6.1). In univariable analysis, we also did not observe associations of hemoglobin level (1.4, 0.4‐5.0), hematocrit level (1.5, 0.4‐5.7), mean corpuscular volume (1.6, 0.2‐15.5), mean corpuscular hemoglobin (0.8, 0.1‐6.7), mean corpuscular hemoglobin concentration (0.2, 0.0‐23.7), platelet count (1.1, 0.8‐1.7), mean platelet volume (2.0, 0.2‐19.7), and lymphocyte count (1.0, 0.7‐1.3) with risk of ATE. In contrast, a higher red cell distribution width (4.4, 1.4‐14.1), leukocyte count (1.2, 1.1‐1.5), and neutrophil count (1.6, 1.1‐2.3) predicted for an increased risk of ATE. After adjustment for age, sex, and smoking the association between the elevated neutrophil count and the ATE risk prevailed (adjusted [adj.] SHR per doubling: 1.6, 95% CI: 1.1‐2.4), while it did not for red cell distribution width (1.7, 0.4‐7.6) and leukocyte count (1.2, 1.0‐1.4). All univariable and multivariable associations between blood count parameters and the risk of ATE are given in Table 2. Univariable associations between complete blood count parameters and different types of ATE events (i.e. myocardial infarction, ischemic stroke, peripheral arterial occlusion) are given in Table S1.

**Table 2:** Association of blood count parameters, cholesterol, d‐dimer, and sP‐selectin levels with the risk of ATE occurrence in patients with cancer

Parameter	Univariable SHR per doubling for ATE risk (95% CI)	Multivariable SHR per doubling for ATE risk^a^ (95% CI)
Erythrocyte count, 10^12^/L	1.5 (0.4‐6.1)	1.7 (0.4‐7.6)
Hemoglobin, g/dL	1.4 (0.4‐5.0)	1.6 (0.4‐5.9)
Hematocrit, %	1.5 (0.4‐5.7)	1.7 (0.5‐6.2)
Mean corpuscular volume, fL	1.6 (0.2‐15.5)	1.6 (0.3‐9.7)
Mean corpuscular hemoglobin, pg	0.8 (0.1‐6.7)	1.0 (0.1‐7.8)
Mean corpuscular hemoglobin concentration, g/dL	0.2 (0.0‐23.7)	0.5 (0.0‐71.5)
Red cell distribution width	4.4 (1.4‐14.1)	2.5 (0.6‐10.0)
Platelet count, 10^9^/L	1.1 (0.8‐1.7)	1.3 (0.9‐2.0)
Mean platelet volume, fL	2.0 (0.2‐19.7)	1.7 (0.2‐17.1)
Leukocyte count, 10^9^/L	1.2 (1.1‐1.5)	1.2 (1.0‐1.4)
Neutrophil count, 10^9^/L	1.6 (1.1‐2.3)	1.6 (1.1‐2.4)
Lymphocyte count, 10^9^/L	1.0 (0.7‐1.3)	1.1 (0.8‐1.4)
Cholesterol level, mg/dL	0.7 (0.3‐1.5)	1.0 (0.4‐2.4)
d‐Dimer level, μg/mL	1.1 (0.9‐1.4)	1.1 (0.9‐1.4)
sP‐selectin level, ng/mL	1.9 (1.3‐2.7)	1.8 (1.2‐2.8)

Note

Subdistribution hazard ratios are calculated per doubling of the parameter. In detail, 1‐unit increase of the log2‐transformed variable.

Abbreviations: ATE, arterial thromboembolism; CI, confidence interval; *n*, number of patients; SHR, subdistribution hazard ratio; sP‐selectin, soluble P‐selectin; VTE, venous thromboembolism.

Adjusted for age, sex, and smoking.

**Figure 1:**
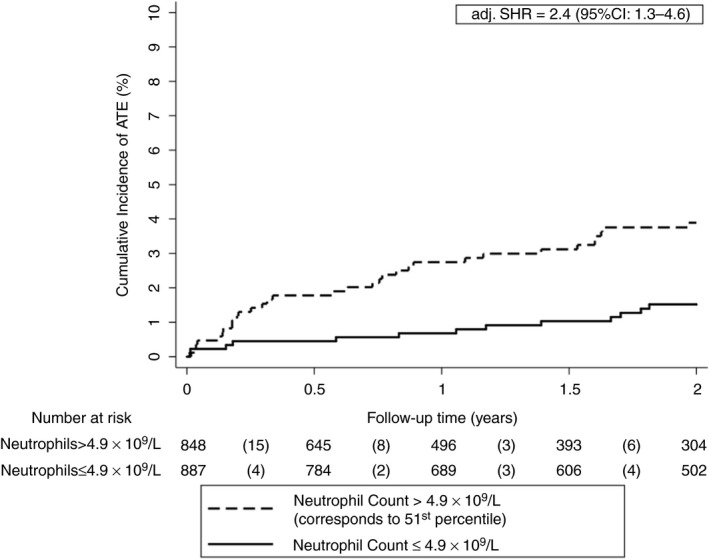
Cumulative incidence of ATE accounting for competing risk (i.e. death and VTE) according to neutrophil levels. The optimal cutoff was determined using Youden′s index. After adjusting for age, sex, and smoking, patients with absolute neutrophil levels above the cutoff (>4.9 × 10^9^/L) had a higher risk of ATE than patients with levels below the cutoff. Note the scaling of the *y*‐axis ranges from 0% to 10% of ATE risk. Neutrophil levels were available in 1735 patients and missing in 148 patients. ATE, arterial thromboembolism; adj. SHR, adjusted subdistribution hazard ratio

After adjustment for age, sex, and smoking no association was found between neutrophil levels dichotomized at the 75th percentile and the risk of ATE in patients with cancer (SHR: 1.4, 0.8‐2.7). However, according to Youden′s index of the receiver operating curve the optimal cutoff point for the neutrophil count was at the 51st percentile (>4.9 × 10^9^/L). The 6‐month, 12‐month, and 24‐ month ATE probability were 1.8 (95% CI: 1.0‐2.9), 2.7% (1.8‐4.0), and 3.9% (2.7‐5.4) in patients with neutrophil levels above this cutoff, and 0.5% (0.2‐1.1), 0.7% (0.3‐1.4), and 1.5% (0.9‐2.5) in patients with neutrophil levels below this cutoff, respectively (adj. SHR: 2.4, 1.3‐4.6, Figure 1).

### 3.4 Association of cholesterol, d‐dimer, and sP‐selectin levels with risk of ATE

In a competing risk regression analysis neither the cholesterol level (SHR per doubling: 0.7, 95% CI: 0.3‐1.5) nor the D‐dimer level (1.1, 0.9‐1.4) was associated with ATE risk. However, an association between elevated sP‐selectin levels and an increased risk of ATE was observed (1.9, 1.3‐2.7). This association remained after adjustment for age, sex, and smoking (adj. SHR per doubling: 1.8, 1.2‐2.8). All univariable and multivariable associations between different blood parameters and the risk of ATE are reported in Table 2. Univariable associations between blood parameters and different types of ATE events are given in Table S1.

**Figure 2:**
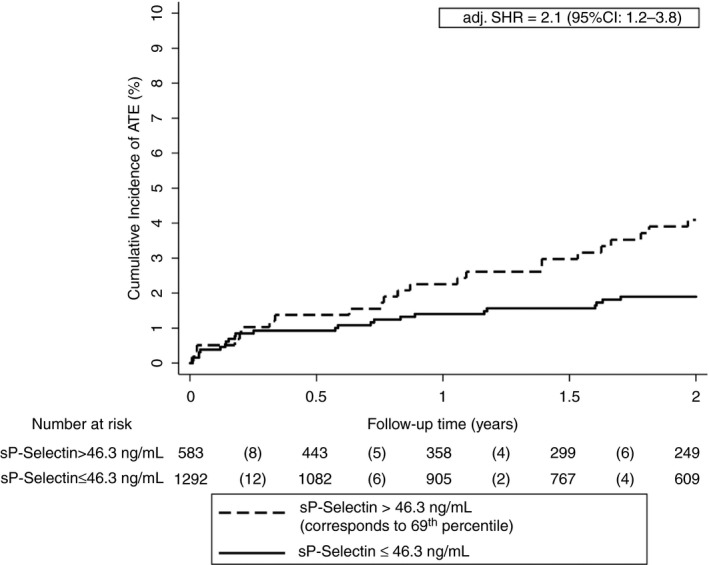
Cumulative incidence of ATE accounting for competing risk (i.e. death and VTE) according to sP‐selectin levels. The optimal cutoff was determined using Youden′s index. After adjusting for age, sex, and smoking, patients with sP‐selectin levels above the cutoff (>46.3 ng/mL) had a higher risk of ATE than patients with levels below the cutoff. Note the scaling of the *y*‐axis from 0% to 10% of ATE risk. sP‐selectin levels were available in 1875 patients and missing in 8 patients. adj. SHR, adjusted subdistribution hazard ratio; ATE, arterial thromboembolism; soluble P‐Selectin, sP‐selectin

After adjustment for age, sex, and smoking no association was found between sP‐selectin levels dichotomized at the 75th percentile and the risk of ATE in patients with cancer (adj. SHR: 1.7, 0.9‐3.1). According to Youden′s index, the optimal cutoff for sP‐selectin was at the 69th percentile (>46.3 ng/mL). The 6‐month, 12‐month, and 24‐month ATE probability were 1.4 (0.7‐2.6), 2.3% (1.3‐3.7), and 4.1% (2.7‐6.0) in patients with sP‐selectin levels above, and 0.9% (0.5‐1.6), 1.4% (0.9‐2.12, and 1.9% (1.3‐2.8) in patients below this cutoff, respectively (adj. SHR: 2.1, 1.2‐3.8, Figure 2).

## 4 DISCUSSION

In this observational cohort study we analyzed the association of complete blood count (CBC) parameters, cholesterol, d‐dimer, and sP‐selectin levels with the risk of ATE in patients with cancer. The major finding of our study was that an elevated neutrophil count and high sP‐selectin levels are associated with the risk of ATE in patients with cancer after correction for age, sex, and smoking. Furthermore, a higher red cell distribution width and an elevated leukocyte count were associated with an increased risk of ATE in univariable analyses. However, these associations did not prevail in multivariable analyses.

First, we examined the association of CBC parameters with the risk of ATE in patients with cancer. Prior studies identified some CBC parameters (e.g. leukocytes, mean platelet volume, red cell distribution width) as biomarkers for arterial and venous thromboembolism in patients with and without cancer.^11^, ^44^, ^45^, ^46^, ^47^, ^48^ As total cholesterol level is a well‐established risk factor for ATE in non‐cancer patients, we decided to investigate its association with ATE in patients with cancer. In addition, we examined whether d‐dimer and soluble P‐selectin (sP‐selectin) levels ‐ two well‐known biomarkers for prediction of cancer‐associated VTE ‐ are also associated with the occurrence of ATE in patients with cancer.^26^, ^31^, ^37^, ^49^, ^50^

Our findings regarding the CBC parameters are partially in line with previous studies. The association of the red cell distribution width with risk of ATE was previously reported in non‐cancer patients. In univariable analysis, we observed an association of the red cell distribution width with the risk of ATE in patients with cancer; however, this association did not prevail in our study cohort after adjustment for age, sex, and smoking.^48^, ^51^, ^52^ Interestingly, an elevated absolute neutrophil count was associated with an increased risk of ATE in patients with cancer after adjustment for age, sex, and smoking, which is in line with prior studies in non‐cancer patients.^53^ However, the total leukocyte count did not predict ATE risk in patients with cancer. This result is in line with a study by Horne et al, who observed that the neutrophil count has a greater ability to predict myocardial infarction than the total leucocyte count.^54^ Since an association between leukocytosis and ATE in the general population has been reported since the 1970s, we cannot exclude the possibility of a false‐negative result in our study.^55^, ^56^ Furthermore, despite the large sample size of our study, the number of arterial events was still relatively low to draw firm conclusions.

It is well known that the higher cholesterol levels are associated with cardiovascular diseases. Therefore, we investigated the association of total cholesterol levels and the risk of ATE in patients with cancer. Against our expectations, we did not find an association of total cholesterol level and ATE in our study population. However, usually those patients with higher body mass index (BMI) have higher cholesterol levels. As weight loss occurs frequently in patients with cancer, we have to consider that these patients would have had a lower absolute level of cholesterol. Furthermore, the measurement of other lipid levels that are known to be associated with the risk of ATE in the general population (e.g. low‐density lipoprotein, high‐density lipoprotein) could provide greater discrimination in estimation of ATE risk in patients with cancer.^57^

Activated platelets play a key role in the development of arterial thrombosis.^58^, ^59^, ^60^ There is evidence that cancer is able to activate platelets, which might trigger thrombosis in at least some cancer types.^61^, ^62^, ^63^, ^64^ Soluble P‐selectin is a well‐established biomarker of platelet and endothelial activation, two processes that are well known for their contribution to the development of cardiovascular diseases..^65^, ^66^ Prior studies by our group showed that higher sP‐selectin is associated with the risk of VTE in patients with and without cancer.^26^, ^31^, ^50^ Furthermore, studies in animal models demonstrated that an inhibition of sP‐selectin can contribute to the resolution of venous thrombosis.^67^, ^68^

Here we observed that sP‐selectin is associated with the risk of ATE in patients with cancer, which is in line with prior studies that have shown that high sP‐selectin levels are associated with an increased risk of ATE in patients without cancer.^27^, ^28^, ^29^ Cancer patients have higher sP‐selectin levels, 69, 70 and in an analysis of the Multi‐Ethnic Study of Atherosclerosis (MESA), Bielinski et al reported that high sP‐selectin levels predicted for an 1.8‐fold increase in the risk of coronary heart disease in non‐cancer patients.^65^ Consistent with this data in the non‐oncologic setting, we observed a similar 1.9‐fold increased relative risk for ATE in cancer patients with elevated sP‐selectin levels. Taken together, the activation of platelets induced by cancer cells might contribute to the increased risk of ATE in patients with cancer, and sP‐selectin reflecting platelet activation might predict ATE in patients with cancer.

Major strengths of our study are the large sample size and the inclusion of patients with a variety of cancer types. Further strengths are the observational design and the adjudication of endpoints by a panel of experts. However, there are also some limitations that need to be addressed. Despite its large sample size, the study could be underpowered, because of the low absolute number of arterial events. In the retrospective study by Navi et al the incidence rate of ATE was 4.7% in patients with cancer compared to 2.2% in control patients.^8^ In comparison the risk of ATE was lower in our study with only 2.6%. As higher age is an important risk factor for the development for ATE we have to take into account the age difference between these two studies. The median age in our study cohort was lower than in the study by Navi et al (61 years vs. 74 years).^7^, ^8^ However, the incidence of ATE in patients with cancer could be underestimated in general, because physicians may be reluctant to perform diagnostic coronary angiographies in cancer patients with poor performance status. Furthermore, we cannot guarantee that all events that might have occurred in the study cohort were captured in our study. A larger study or the identification of more arterial events via routine screening may have yielded higher statistical power and increased sensitivity and thereby enabled the identification of more potential biomarkers for ATE risk prediction in patients with cancer. This hypothesis is supported by the fact that we had to choose cutoffs based on a receiver‐operating characteristic in addition to the empirically chosen cutoff at the 75th percentile to reach statistical significance in between‐group comparisons. However, cutoffs are well known for their potential to generate false‐positive and false‐negative results. To minimize this potential bias, we focused on continuous log2‐transformed variables in our main analyses. Finally, our results have to be considered hypothesis generating and need to be confirmed in further studies.

Furthermore, we cannot address the effect of inflammation, infection, or other acute diseases on blood parameters and ATE risk. However, we tried to minimize this effect by excluding patients with an overt bacterial or viral infection. As the association of blood parameters and biomarkers with risk of specific types of ATE would be interesting, we have performed subgroup analyses. However, because of the relatively low number of arterial thromboembolic events and the resulting low power, these results must be considered with caution.

With cardiovascular diseases and cancer becoming more prevalent in an aging population, the investigation of the interrelation between these common diseases is of emerging importance.^32^ We have identified parameters that could help in predicting cardiovascular risk in patients with cancer. Further research in larger cohorts is necessary to validate our finding that the absolute neutrophil count and the sP‐selectin level are associated with an increased risk of ATE in patients with cancer after adjustment for cardiovascular risk factors.

## DATA ACCESS AND RESPONSIBILITY

Ella Grilz and Cihan Ay had full access to all the data in the study and take responsibility for the integrity of the data and the accuracy of the data analysis.

## CONFLICT OF INTEREST

E.G., C.M., O.K., J.R., F.P., W. L., I.M.L., I.P., and C.A. have no conflict of interest to declare.

## AUTHOR CONTRIBUTIONS

Ella Grilz acquired, analyzed, and interpreted data; performed statistical analyses; coordinated the study; and drafted the manuscript; Christine Marosi critically revised the manuscript for important intellectual content and contributed to the study design; Oliver Königsbrügge acquired and interpreted data and recruited patients; Julia Riedl recruited patients and performed experiments; Florian Posch performed statistical analyses and critically revised the manuscript; Wolfgang Lamm critically revised the manuscript for important intellectual content and contributed to the study design; Irene M. Lang critically revised the manuscript for important intellectual content, was a member of the adjudication committee, and contributed to the study design; Ingrid Pabinger designed and conceived the study, obtained funding, critically revised the manuscript for important intellectual content, and provided administrative support. Cihan Ay acquired data, designed and supervised the study, obtained funding, critically revised the manuscript for important intellectual content, and provided administrative support; all authors reviewed and edited the manuscript and finally approved the article.
