# Supplementary material for: Association of complete blood count parameters, d‐dimer, and soluble P‐selectin with risk of arterial thromboembolism in patients with cancer
Source: J Thromb Haemost. 2019 Jun 17;17(8):1335–44. doi: 10.1111/jth.14484 (PMC6771479; doi:10.1111/jth.14484)
Supplement: Supplementary file 1 [file JTH-17-1335-s001.docx]

**Supplementary table 1.**

**Association of complete blood count parameters, d-Dimer and soluble P-selectin with risk of myocardial infarction, ischemic stroke and peripheral arterial occlusion in patients with cancer.**

Univariable Fine & Gray competing risk regression models were used to estimate subdistribution hazards (SHR). Reported are SHRs per doubling of the parameter (i.e. one unit increase of the log2-transformed variable). 95% confidence intervals are given in brackets. Venous thromboembolism and death from any cause were considered competing events.^41^

MCV=mean corpuscular volume; MCH=mean corpuscular hemoglobin, MCHC=mean corpuscular hemoglobin concentration; RDW=red cell distribution width; MPV=mean platelet volume

| **Parameter** | **Myocardial infarction (n=20)** | **Ischemic stroke (n=16)** | | **Peripheral arterial occlusion (n=12)** | |
| --- | --- | --- | --- | --- | --- |
| Erythrocyte count, 10^12^/L | 0.8 (0.1-5.1) | 5.3 (0.8-33.6) | 1.0 (0.0-37.6) | |  |
| Hemoglobin, g/dl | 0.9 (0.1-6.0) | 3.9 (0.6-26.2) | 0.8 (0.0-13.6) | |  |
| Hematocrit, % | 0.8 (0.1-5.0) | 4.6 (0.9-23.7) | 1.2 (0.0-28.0) | |  |
| MCV, fL | 1.4 (0.1-16.9) | 1.4 (0.1-17.4) | 2.2 (0.0-7945.1) | |  |
| MCH, pg | 1.1 (0.1-21.6) | 0.9 (0.0-22.2) | 0.4 (0.0-60.0) | |  |
| MCHC, g/dL | 1.3 (0.0-4937.5) | 0.5 (0.0-6409.9) | 0.0 (0.0-3.6) | |  |
| RDW | **6.2 (1.1-33.2)** | 1.3 (0.1-15.0) | **8.3 (1.4-49.9)** | |  |
| Platelet count, 10^9^/L | 0.9 (0.5-1.4) | 1.7 (0.9-3.5) | 1.1 (0.5-2.6) | |  |
| MPV, fL | 2.1 (0.1-73.3) | 3.5 (0.1-216.3) | 1.0 (0.0-57.5) | |  |
| Leukocyte count, 10^9^/L | 1.1 (0.8-1.5) | **1.4 (1.2-1.6)** | 1.3 (1.0-1.9) | |  |
| Neutrophil count, 10^9^/L | 1.4 (0.7-2.7) | **2.2 (1.3-3.1)** | 1.4 (0.7-2.7) | |  |
| Lymphocyte count, 10^9^/L | 1.2 (0.9-1.5) | 0.9 (0.5-1.7) | 0.7 (0.4-1.2) | |  |
| Cholesterol level, mg/dL | 1.1 (0.2-4.8) | 0.4 (0.2-1.1) | 0.7 (0.1-4.2) | |  |
| D-dimer level, μg/mL | 1.2 (0.8-1.6) | 1.3 (1.0-1.8) | 0.8 (0.6-1.2) | |  |
| sP-selectin level, ng/mL | **2.1 (1.2-3.6)** | **2.5 (1.3-4.8)** | 1.0 (0.5-1.8) | |  |
